# Exploiting genomics to mitigate the public health impact of antimicrobial resistance

**DOI:** 10.1186/s13073-022-01020-2

**Published:** 2022-02-16

**Authors:** Claire Waddington, Megan E. Carey, Christine J. Boinett, Ellen Higginson, Balaji Veeraraghavan, Stephen Baker

**Affiliations:** 1 Cambridge Institute of Therapeutic Immunology and Infectious Disease, University of Cambridge School of Clinical Medicine, Cambridge Biomedical Campus, Cambridge, CB2 0AW UK; 2 Department of Medicine, University of Cambridge School of Clinical Medicine, Cambridge Biomedical Campus, Cambridge, UK; 3 Wellcome Sanger Institute, Hinxton, Cambridge, UK; 4 Department of Microbiology, Christian Medical College, Vellore, Tamil Nadu India

**Keywords:** Antimicrobial resistance, Public health, Genomics, Surveillance, Vaccines, Diagnostics

## Abstract

Antimicrobial resistance (AMR) is a major global public health threat, which has been largely driven by the excessive use of antimicrobials. Control measures are urgently needed to slow the trajectory of AMR but are hampered by an incomplete understanding of the interplay between pathogens, AMR encoding genes, and mobile genetic elements at a microbial level. These factors, combined with the human, animal, and environmental interactions that underlie AMR dissemination at a population level, make for a highly complex landscape. Whole-genome sequencing (WGS) and, more recently, metagenomic analyses have greatly enhanced our understanding of these processes, and these approaches are informing mitigation strategies for how we better understand and control AMR. This review explores how WGS techniques have advanced global, national, and local AMR surveillance, and how this improved understanding is being applied to inform solutions, such as novel diagnostic methods that allow antimicrobial use to be optimised and vaccination strategies for better controlling AMR. We highlight some future opportunities for AMR control informed by genomic sequencing, along with the remaining challenges that must be overcome to fully realise the potential of WGS approaches for international AMR control.

## Background

Antimicrobial resistance (AMR) is one of the greatest current threats in international public health [1]. The rapid increase and worldwide spread of AMR threaten the advances in modern medicine, compromising the treatment of common infections such as pneumonia, urinary tract infections, and tuberculosis as well as the care of patients needing organ transplantation, complex surgery, cancer chemotherapy, and intensive care [2]. There are also significant economic and societal costs associated with AMR infections [3, 4]. These costs are attributed with longer hospital stays, higher medical bills, and increased mortality. Although AMR is a global problem, the burden of AMR falls disproportionately on low- and middle-income countries (LMICs), where it threatens sustainable development [4–6].

The first wave of antimicrobials was derived from naturally occurring compounds; therefore, the emergence of AMR is largely a natural process, and AMR genes have been detected in samples originating millions of years before the widespread use of antimicrobials [7–9]. However, an exponential growth in antimicrobial use in recent decades has exerted enormous selective pressures on bacterial populations, which has dramatically accelerated the evolution of AMR [10]. Indeed, as soon as mankind develops a new class of small molecules to kill bacteria, organisms evolve to resist their action, leading to an increasing prevalence of multi-drug-resistant (MDR), extended-drug-resistant (XDR), and pan-drug-resistant (PDR) organisms.

The detection of AMR has been traditionally reliant on culture-based antimicrobial susceptibility testing (AST), which remains the mainstay of clinical microbiology and patient management. Whilst phenotyping provides direct visual evidence of how a bacterium will interact with an antimicrobial, it generally provides little or no data regarding the resistance mechanisms, with disparate genetic clones often demonstrating identical resistance profiles [11]. Genetic typing methods, such as multi-locus sequence typing (MLST), provide a higher level of pathogen resolution compared to AST but are highly restrictive as they only describe a small fraction of a genome. Whole-genome sequencing (WGS), in contrast, provides genome-wide information at the single nucleotide level that can be used to identify the presence and mechanisms of AMR, as well as pathogen identity, virulence, and ancestry [12–14]. The advent of next-generation sequencing (NGS), via high-throughput, parallel sequencing of DNA fragments, has allowed pathogen genomes to be determined rapidly and at comparatively low cost [13, 15, 16]. The universality of the genetic code allows a unified approach to be applied to all organisms, and a range of technologies and platforms can provide the same data output [11]. Comparative phylogenetic analysis can be exploited to determine the degree of relatedness between different isolates based on the extent of the similarity between genomes and, when overlaid with epidemiological and clinical data, can inform our understanding of the specific temporospatial dynamics of AMR and transmission [11]. Additionally, the recent optimisation of metagenomic sequencing approaches circumvents the necessity for culture entirely. Therefore, by incorporating all available genomic material in a sample, metagenomic analysis facilitates a shift in focus from an individual pathogen to the community, microbiome landscape, generating a highly detailed model of how pathogens interact, and how they mobilise and access AMR genes [17].

The power of WGS is being increasingly employed to address the public health challenge of AMR, supporting surveillance, outbreak investigation, and contributing to improved diagnostics and therapeutics as highlighted in Table 1 [12, 27]. This review highlights some of the successes and advances supported by WGS in these areas and outlines future directions and remaining challenges associated with using WGS technologies to support public health efforts for AMR.

**Table 1:** Use cases for whole-genome sequencing (WGS) in mitigating the public health impact of antimicrobial resistance (AMR)

Trigger	Uses of WGS/workflow	Main findings	Advantages of using WGS
**Use case 1: International surveillance—determining the population structure and epidemiology of carbapenem-resistant** ***K. pneumoniae*** **(CR-Kp) across Europe** **[**18**]**			
Primary reservoirs and transmission dynamics of CR-Kp across Europe are incompletely understood.	Hospital laboratories across Europe submitted consecutive clinical isolates of CR-Kp, along with a comparator susceptible isolate for sequencing.	CR-Kp largely resulted from carbapenemase acquisition; nosocomial acquisition was the main cause of CR-Kp spread.	Provided a benchmark for ongoing surveillance of CR-Kp. Highlighted the role of nosocomial spread.
**Use case 2: Enhancing the national surveillance of antimicrobial resistance in the Philippines** **[**11**]**			
National laboratory-based surveillance had shown increasing AMR prevalence over the10 years previously, but understanding of the epidemiology and drivers of AMR were lacking.	WGS capability was introduced to the existing surveillance programme. Retrospective sequencing of MDR GNB obtained prior to the introduction was undertaken and analysed with phenotypic and epidemiological data to provide baseline data and inform control measures.	Drivers of carbapenem resistance at different levels of the healthcare system were identified, including a localised outbreak of plasmid-driven CR-Kp affecting a specific hospital, through the detection of the introduction and country-wide spread of a high-risk epidemic clone, *E. coli* ST410.	Detailed understanding of the epidemiology and drivers of AMR enabled the introduction of effective infection control measures. Data were contributed to international AMR surveillance efforts, improving the global coverage.
**Use case 3: Investigating an MRSA outbreak in a neonatal unit** **[**19**]**			
Phenotypically similar MRSA isolates were identified from patients on a neonatal unit over a 6-month period but could not be linked temporally or geographically, suggesting that the full extent of the outbreak had not been identified.	All MRSA isolates obtained from patients on the neonatal unit over a 6-month period underwent WGS regardless of phenotypic characteristics. MRSA isolates with antibiograms similar to the outbreak strain, identified from the community, and screening samples taken elsewhere in the hospital were also sequenced.	Two previously excluded isolates were identified as being part of the outbreak by phylogenetic analysis, allowing temporal links between cases to be established. A wide transmission network beyond the neonatal unit was identified.	WGS allowed a large number of isolates to be tested and related strains to be accurately identified, thereby enabling full outbreak reconstruction. Combining WGS data with clinical and epidemiological data enabled the identification of outbreak source and successful instigation of infection control measures.
**Use case 4: Investigating the direction of transmission in an** ***A. baumannii*** **outbreak in a UK hospital** **[**20**]**			
Molecular typing of a cluster of *A. baumannii* isolates obtained in a UK hospital suggested a clonal outbreak, but the chain of transmission between cases could not be established from the existing laboratory, clinical, and epidemiological data.	A cluster of isolates obtained from patients with identical molecular typing profiles and antibiograms underwent WGS analysis to inform the understanding of the direct transmission between patients.	Phylogenetic analysis enabled the identification of the index case and the subsequent chain of transmission to be determined. One patient/isolate was shown to be unrelated and was excluded from the outbreak investigation.	WGS-enabled directionality of transmission can be determined, allowing accurate reconstruction of the outbreak.
**Use case 5: Contact tracing and detection of secondary cases of TB** **[**21**]**			
Screening and detection of secondary cases of TB are essential for TB control. Accurate identification of case clusters and transmission networks is hampered by the limited resolution provided by molecular typing.	Clinical TB isolates in the Netherlands in 2016 were analysed by both molecular typing and WGS. The degree of discrimination and accuracy in identifying potentially related cases was compared between the two methods.	WGS was better able to decimate the relatedness of isolates, clustering a smaller proportion of isolates as related compared with molecular typing (25% vs. 14%) and increasing proportion confirmed as epidemiologically linked (57% vs. 31%).	WGS facilitated the identification of transmission events, facilitating contact tracing as well as informing the wider understanding of TB control.
**Use case 6: Identifying the drivers of AMR in atypical enteropathogenic** ***E. coli*** **(aEPEC) strains isolated from children < 5 years in four sub-Saharan African countries and three South Asian countries** **[**22**]**			
The frequency, mechanisms, and drivers of AMR in intestinal isolates of *E. coli* in children in the community in many countries worldwide were unknown.	Phenotypic susceptibility and WGS of isolates were analysed and correlated with antimicrobial use, disease status (symptomatic/asymptomatic), phylogenetic lineage, and geographic location.	High rates of AMR were shown, with 65% of isolates resistant to at least 3 antimicrobial drug classes. A diverse range of genetic mechanisms of AMR was shown, with geographic location and the associated antimicrobial use pattern being the strongest predictors of AMR.	WGS was used to provide a detailed analysis of AMR across a large geographical area, providing insights into the AMR epidemiology, spread, and drivers.
**Use case 7: Investigating colistin resistance detected in commensal** ***E. coli*** **in food stock animals in China** **[**23**]**			
Routine surveillance had detected a sharp increase in the rates of colistin resistance in colonising bacteria from pigs in China, but the mechanism of this resistance was not known.	Conjugation experiments were undertaken to confirm the presence of plasmid-associated, transmissible colistin resistance. WGS of the plasmids was used to identify the gene responsible.	The sequence of the plasmid-associated colistin resistance gene was identified and designated *mcr-1.*	The genetic basis of a new, AMR mechanism was identified and described, allowing ongoing surveillance, as well as informing investigation and detection of this emerging threat in other settings.
**Use case 8: Detecting of transmitted drug resistance (TDR) in newly diagnosed, treatment naïve HIV-1-positive patients** **[**24**]**			
Multiple genetic mutations contribute to HIV drug resistance. Mutations often only affect a fraction of the viral population in any given patient (low-level variants), but, if present in combination with other mutations or at key sites, likely contribute to treatment failure. Existing methods are insufficiently sensitive to detect low-level variants that can compromise treatment.	NGS was used to detect TDR, including low-level variants affecting ≥ 2% or more of the viral population, in treatment-naïve, clinical trial participants enrolled across 35 countries worldwide with a HIV viral load > 1000 copies/ml.	NGS revealed many low-level variants that were undetected by existing methods. Significant geographic diversity was seen in the prevalence of different TDR mutations.	NGS provided a more comprehensive assessment of TDR prevalence in individual patients, and in different regions of the world, helping guide empiric treatment choice and understanding of clinical outcomes in different patients and settings.
**Use case 9: Understanding the epidemiology of MDR and XDR pathogens amenable to control by vaccination** **[**25**,** 26**]**			
AMR is increasingly threatening the success of treatment for typhoid fever. Resistance to the last effective oral agent, azithromycin, was detected in Bangladesh and subsequently in Pakistan, but the genetic mechanism and the likely hood of dissemination were unknown.	Clinical isolates of azithromycin-resistant *S.* Typhi were analysed by WGS. The phylogenetic analysis enabled the contextualisation of the strains within contemporaneous *S*. Typhi isolates in both settings.	Phylogenetic analysis showed that resistant isolates in Bangladesh and Pakistan resulted from the independent acquisition of mutations in the same gene highlighting the extent of selection pressure on azithromycin and the imperative need for disease control by vaccination.	WGS was used to identify and investigate two separate outbreaks of azithromycin-resistant *S.* Typhi. These data helped provide the impetus to roll out novel typhoid conjugate vaccines to control infection.

## AMR surveillance

Surveillance is the cornerstone of public health efforts in controlling AMR. AMR surveillance has traditionally relied on phenotypic AST, but different testing methods, variation in interpretation, the extent to which thresholds are clinically validated, and changes in interpretive guidelines limit standardisation. WGS data overcomes many of these limitations, providing detailed insights that can greatly augment the value of AMR surveillance. Such data can inform an understanding of AMR evolution and spread, inform control strategies, facilitate the detection of new and emerging threats, and support new diagnostic and therapeutic approaches [4, 10, 12, 28–30].

Underlying the expansion of AMR is a dynamic and complex interaction between microbes, AMR encoding genes, and mobile genetic elements that act as vehicles for AMR via horizontal gene transfer (HGT) [7, 10, 11, 31]. Once horizontally transferred AMR genes have become chromosomally integrated, clonal expansion can lead to the rapid dissemination of these genes. This phenomenon has been observed with methicillin-resistant *Staphylococcus aureus* (MRSA), penicillin-resistant *Streptococcus pneumoniae*, vancomycin-resistant enterococci, and fluroquinolone-resistant *Clostridium difficile* [11, 32–37]. The granular resolution afforded by WGS allows inferences on the nature of AMR evolution and dissemination [38], providing insights that can help contain AMR and protect public health.

### International surveillance

Increased accessibility to WGS has significantly enhanced our understanding of the global evolution and spread of AMR. High-throughput WGS methods have facilitated the sequencing of large, geographically representative collections of isolates, overcoming many of the biases associated with historic, small-scale studies that were often skewed by the domination of local clonal dissemination. This paradigm is illustrated by a modified understanding of AMR in *E. coli* ST131, which has rapidly spread to become a frequent cause of healthcare and community-acquired infection since it was first described in 2008 [33]. ST131 *E. coli* frequently exhibit cephalosporin (most commonly due to a CTX-M-15 encoding gene) and fluroquinolone resistance [33, 39]. The development of AMR in ST131 *E. coli* was initially speculated to have arisen from frequent and independent acquisitions of mobile genetic elements [40]. However, a study from the USA concluded that the success of AMR in ST131 *E. coli* was associated with a sustained clonal expansion. However, it was not until a more comprehensive global study that the true diversity of AMR in ST131 was revealed, which identified the chromosomal integrations of various resistance genes, the persistence and evolution of mobile elements within sub-lineages, and the sporadic acquisition of several different resistance elements [41]. As well as enhancing the mechanistic understanding of AMR, this study highlighted the need for multifaceted control strategies that could limit the spread of ST131, as well as the spread of mobile genetic elements to other pathogens [42]. These studies were foundational for additional work specifically investigating risk factors for ST131 infection and spread. ST131 is now known to be a common gut commensal, with opportunistic infections occurring mainly in functionally compromised hosts such as the elderly, particularly those having prior antimicrobial use and living in long-term care facilities [43–47]. Infection control measures for ST131 *E. coli* in long-term care facilities focusing on contact precautions have been largely ineffective [45, 48], leading to the strategy of limiting multi-bedded rooms and communal dining facilities [49]. The targeted screening of hospitalised patients with specific risk factors for ST131 facilitated patient isolation to prevent nosocomial transmission, as well as better management of empirical broad-spectrum antimicrobial therapy in specific high-risk patient groups [46, 47].

Global AMR surveillance has been bolstered by programmes such as the Global Antimicrobial Resistance and Use Surveillance System (GLASS) from the World Health Organization (WHO) [4], and the European Antimicrobial Resistance Surveillance Network (EARS-Net) [50]. These networks encourage data sharing and provide standardised templates for reporting AST data to facilitate comparative analysis. Recently, such networks have documented a rapid increase in MDR Gram-negative bacteria (GNB) that have become recognised as leading AMR threats, including third-generation cephalosporin-resistant and carbapenem-resistant *Enterobacterales* (CRE), carbapenem-resistant *Acinetobacter baumannii* and *Pseudomonas aeruginosa*, [1, 22, 51–55]. These networks are expanding their evidence base for other key threats, such as *Klebsiella pneumoniae*, which can cause a wide range of infections, including pneumonia, bacteraemia, and urinary tract infections [56], and have been identified as a crucial entry point for MDR into *Enterobacterales* [57–59]*.* Carbapenem-resistant *K. pneumoniae* (CR-Kp) is considered to be the fastest-growing AMR threat in Europe [60] and has a high attributable mortality rate (estimated as 30–70%) [61, 62]. Systematic surveillance of CR-Kp across Europe demonstrated that nosocomial spread was driving this epidemic, with carbapenemase acquisition occurring across diverse phylogenetic backgrounds [18]. As well as describing the epidemiology of CR-Kp, this study found that isolates were concentrated into four clonal lineages: sequence types (ST) 11, 15, 101, and 258/512. These data contrast with studies in other settings that found high genomic plasticity in CRE, with frequent poly-clonal and poly-species horizontal gene transfer accounting for much of the acquired resistance [54, 63, 64]. This inconsistency highlights the complex interplay between bacteria and MDR genes, with the role of clonal expansion [18] and HGT [54, 63, 64] potentially contributing variably across different timeframes and geographies.

Embedding WGS into prospective, cross-national surveillance will further strengthen AMR surveillance, as highlighted by the recent outbreak genomic investigation of *bla*_NDM-1_ and *bla*_OXA-48_ CR-Kp in Germany [65]. In the absence of routine collection and reporting of European-wide WGS data, investigators pooled data from 13 national surveillance systems to investigate the spread of the CR-Kp outbreak, identifying cross-border transmission of several unrelated clusters of *bla*_NDM-1_- and *bla*_OXA-48_-positive CR-Kp. It is anticipated that the planned incorporation of WGS into the European Antimicrobial Resistance Genes Surveillance Network (EURGen-Net) will facilitate the routine detection of such transmission events and inform targeted control measures [65].

### National surveillance

The inclusion of WGS can similarly strengthen national AMR surveillance. The introduction of WGS into AMR surveillance in the Philippines, for example, showed that HGT was key for driving carbapenem resistance in *Klebsiella*, with mobile genetic element (MGE) acquisition often leading to MDR [11]. WGS data also identified a localised plasmid-driven outbreak of CR-Kp, where various infection control measures, including patient isolation, were initiated [11]. By contributing WGS data to the WHO international surveillance network, national surveillance data also enhanced the global understanding of some high-risk clones of interest, notably ST147 CR-Kp, of which a previously uncharacterised clade was described [11].

National surveillance can directly guide the need for AMR control measures that can be driven, resourced, and monitored centrally, as was demonstrated in Israel following the introduction of CR-Kp ST258 in 2005. The initial measures were unsuccessful at controlling the spread of CR-Kp ST258, and infections rapidly rose to a peak incidence of 41.9 per 100,000 patient-days [61]. Nationally mandated control measures were subsequently introduced in 2007, successfully reducing the incidence of infections by 79%, as well as the rate of asymptomatic carriage [52, 66–69].

The routine inclusion of WGS into national surveillance can also be used to evaluate the risk from emerging AMR threats, such as transferable colistin resistance [70]. Phenotypic colistin resistance testing is not routinely performed and therefore is not readily detectable without WGS [70]. Routine WGS of GNB in the UK generated a substantial data resource over time, which was mined to determine the extent of the spread of plasmid-encoded colistin resistance after it emerged as an AMR threat [70]. Although colistin resistance can be detected in organisms from a range of environments, this retrospective study found that it remained uncommon in the UK, leading to the monitoring of colistin prescribing in a bid to minimise any potential selective pressure within the UK.

### Local surveillance

Healthcare facilities house patients with serious and complex infections in proximity to patients with compromised immunity, in settings that are characterised by high antimicrobial use [71, 72]. In this context, healthcare-associated infections (HCAIs), frequently with pathogens that are highly drug-resistant, are a significant challenge requiring rigorous infection prevention and control practices to reduce their incidence [72]. The ability to perform WGS in a clinically relevant turnaround time has made genomics an actionable front-line tool to investigate HCAIs [73].

A key strength of WGS is that it permits the degree of relatedness between selected isolates to be determined and, in the context of HCAIs, allows the reconstruction of nosocomial outbreaks [14, 39]. One of the earliest applications of this process was in a retrospective investigation of a protracted outbreak of MRSA in a neonatal unit [73]. By reconstructing the full outbreak, the source was eventually identified, ultimately, supporting infection control measures that eventually led to its resolution [19]. Other studies of *S. aureus* HCAI have similarly shown the improved granularity of WGS compared to other approaches, with more conventional *S. aureus*-specific staphylococcal protein A (*spa*) typing failing to detect transmission events, as well as the false attribution of unrelated isolates as HCAIs [74].

By exploiting a ‘molecular clock’ (the mutation rate in the genome of a specific organism), WGS can also infer directionality in outbreaks [13, 14, 19, 27]. This approach was key to understanding an outbreak of MDR *Acinetobacter baumannii* in a UK hospital treating civilian patients and injured military personnel returning from the Middle East [20]. Pre-admission colonisation of the wounds of military personnel with distinct lineages of *A. baumannii* was described, with the onward transmission of one specific clone leading to the infection of a civilian. Importantly, an additional civilian infection was not associated with the military-related infection, highlighting that other distantly related *A. baumannii* were also circulating. Similarly, the utility of WGS in determining the chains of transmission has also been demonstrated for MRSA and CR-Kp [75].

By generating detailed insights into HCAI, WGS surveillance can help identify the main sources of AMR, providing an opportunity for targeted infection control measures that can reduce the risk of further transmission events [73]. A large study of HCAI CR-Kp showed that the majority of inpatient infections resulted from the transfer of a relatively small number of patients from specific, high-risk facilities and that targeting interventions at such facilities could significantly reduce HCAIs across the healthcare system [75]. WGS-based investigations can similarly limit the use of unnecessary or ineffective measures that can be costly to implement. For example, studies of MRSA transmission have shown that patient-to-patient transmission is relatively uncommon and that persistently colonised staff are more frequent sources of infection than patients [74]. Targeting infection control at the staff rather than patients may therefore be a more efficient and more cost-effective control mechanism [76].

Prospectively applying WGS for local HCAI control has also been suggested for early outbreak detection, and thereby early intervention and source control, with a substantial cost saving [4]. This approach may be especially true for problematic pathogens in high-risk settings, such as MDR *K. pneumoniae* in ICUs [77]. WGS of routinely collected MRSA samples, obtained from both hospital and community settings over a year in the US identified extensive and previously unrecognised transmission events, highlighting the utility of routine WGS screening in high-risk patients and/or settings [76].

### Community surveillance and outbreak investigation

WGS is also contributing to community public health efforts, such as contact tracing and the detection of secondary cases of TB. Contact tracing is difficult as there is often a long interval between the initial infection and the diagnosis. Several countries, such as the UK, have established a nationwide WGS database of TB to facilitate the identification of transmission events. This system enhances contact tracing as well as informs a wider understanding of TB control. Using WGS in preference to variable number tandem repeat (MIRU-VNTR) typing has been shown to reduce the number of false case clusters and has improved the detection of cases where potential clusters are identified [21, 78–81].

## Understanding the drivers of AMR

The paradigm that the rapid expansion in AMR is a response to selective pressure from antimicrobial use drives AMR is well accepted [82]. WGS is being used to interrogate this selective evolution at the molecular level, across wide geographical areas, and between genetically diverse organisms. WGS was exploited to investigate AMR and the clinical epidemiology of atypical enteropathogenic *E. coli* isolated from symptomatic and asymptomatic children in South Asia and sub-Saharan Africa [22]. Despite broad geographical, symptomatic, and phylogenetic diversity, 65% of the bacterial isolates were resistant to three or more classes of antimicrobials. In this study, the best predictor of resistance profile was not the presence or absence of clinical symptoms or genetic lineage, but the geographical patterns of antimicrobial usage [22].

On an individual patient level, the likelihood of developing infection with extended-spectrum beta-lactamase-producing bacteria is associated with an increased length of hospital stay prior to infection, exposure to antimicrobials, and recent overseas travel [83]. Predictors of MDR GNB infection more broadly include male sex, older age, and co-morbidities [84]. Changes in the human microbiota occur in response to illness, particularly when associated with frequent and/or prolonged antimicrobial exposure. The *Enterobacterales* are habitual colonisers of the gastrointestinal tract, where they can act as a major reservoir for mobile AMR genes [22]. Metagenomic studies have shown that commensal bacteria in healthy individuals help maintain pathogenic bacteria at a low density, meaning that carriage is rarely problematic [71]. However, when patients undergo an invasive procedure, there is a loss in microbial diversity followed by colonisation with pathogenic bacteria [85]. This effect is can be exacerbated by antimicrobial use, which frequently results in the selection of drug-resistant pathogens [71] and facilitates HGT of AMR genes between bacterial lineages and species [7, 86]. WGS studies are now being used to determine the colonisation factors that facilitate the rapid growth and persistence of certain pathogens in such circumstances, with the hypothesis that therapeutics targeting persistent organisms may be developed to control pathogen colonisation.

### AMR and ‘One Health’

One Health focuses on understanding the interconnectivity between ecosystems [87], recognising that human health is connected with, and dependent on, the health of animals, plants, and the wider environment [4, 17, 88]. AMR transmission occurs both within and across different ecosystems, facilitated by close animal and human contact, food, and water systems, all of which are influenced by culture and economics [87]. Understanding this complex interplay is crucial for the control of AMR. WGS is being increasingly used to support One Health aspects of AMR, such as by interrogating the spread of AMR via food and farming [88, 89], building on an established food-borne disease surveillance [27], and outbreak investigation [90, 91]. A WGS-based investigation of commensal *E. coli* isolates in livestock in China after a rapid increase of colistin resistance, for example, showed that this increase was due to the emergence of a plasmid associated colistin resistance gene in *E. coli*, designated MCR-1, and led to international efforts to control the dissemination of MCR-1 via food supply networks [23].

## Optimising antimicrobial use through WGS-based rapid diagnostic tests

Rapid, accurate, low-cost diagnostic tests can aid in optimising and limiting antimicrobial use, thereby minimising the potential selective pressures. Culture-based microbiological diagnostics and AST are widely utilised for bacterial infections but are not rapid and not applicable to viral infections or fastidious organisms. WGS-based diagnostic approaches are being used to overcome these limitations, notably for HIV and tuberculosis, and offer the prospect of improved outcomes.

The prognosis of HIV infection has been transformed by combination anti-retroviral therapy (cART), but these treatments are complicated by the emergence of viral resistance. Genomic replication of RNA viruses utilises a reverse transcriptase lacking proofreading capacity, leading to the accumulation of mutations, with in host infection existing as a population of closely related genomes (quasispecies) [92]. Further in host genetic variability can result from the recombination of viruses infecting the same cell and the accumulation of variants over time [93]. WGS can detect viral variants at a prevalence of ~ 1%, and WGS-based resistance testing is used prior to initiating cART to detect mutations conferring drug resistance in the infecting quasispecies [94–97]. The full clinical implications of minority variants are unclear [98], but they predict failure of first-line regimens [99] and have been shown to be increasing in some locations [24], highlighting the need for the global monitoring of drug resistance in HIV.

Treatment success in *M. tuberculosis* declines from 83% for susceptible isolates to 54% and 30% for MDR- and XDR-TB isolates, respectively [100]. *M. tuberculosis* is slow growing, with culture-based diagnosis and AST often taking weeks and requiring a high-level biosafety laboratory. Poor reproducibility, uncertainty regarding appropriate drug concentrations for susceptibility testing [101], and a propensity for laboratory contamination can lead to false positives [102–104]. The *M. tuberculosis* genome comprised a single chromosome with drug resistance mediated through mutations in core genes or promoters [105], making the organism perfectly suited to WGS diagnostic approaches [105]. WGS diagnostics significantly decrease the time to a confirmatory TB diagnosis [106, 107], plus it reliably predicts the drug resistance profile [108], whilst facilitating the detection, monitoring, and diagnosis of de novo drug resistance [109]. These approaches have revolutionised TB control and are being increasingly used routinely in TB management.

## Informing vaccination strategies to control AMR

Vaccination has an established role in AMR reduction. Conjugate vaccines for *Haemophilus influenzae* and *Streptococcus pneumoniae* have been shown to not only decrease the incidence of disease, but also reduce AMR [110, 111]*.* Increasing access to these vaccines would likely have a further impact on decreasing both disease and AMR [112, 113]. Vaccines against viral infections including influenza, rotavirus, and varicella zoster have also been shown to reduce antimicrobial use by decreasing the incidence of secondary bacterial infections and syndromic presentations for illnesses that would lead to antimicrobial use [112–114]*.*

More recently, vaccination has been used as a direct mechanism for controlling AMR in typhoid fever, the result of many years of effort at understanding typhoid fever, in which WGS has played a significant role. Typhoid fever is caused by the bacterium *Salmonella enterica* serovar Typhi (*S*. Typhi) and causes an estimated 10.9 million cases and 116,800 deaths annually [115]. Whilst most cases of typhoid fever can be treated with antimicrobials, the emergence and spread of AMR have posed an increasing threat to typhoid control, particularly in South Asia, where resistance to each class of oral antimicrobials used to treat typhoid fever has emerged [25]. *S*. Typhi is highly clonal [116], and MDR *S*. Typhi is heavily associated with a single haplotype (H58) [117]. The global spread of H58 has largely replaced other non-H58 haplotypes and is causing sustained ongoing typhoid transmission in east and southern Africa [117]. MDR within H58 is linked with a single mobile element that was introduced via a specific plasmid (IncHI1-PST6) but has since transferred to the chromosome. As new resistance phenotypes have emerged, WGS has facilitated the further investigation of the underlying molecular mechanisms, including the recent emergence of azithromycin resistant typhoid in Bangladesh, Pakistan, Nepal, and India [26, 118, 119]. WGS-based investigation of an outbreak of ceftriaxone-resistant typhoid in Hyderabad, Pakistan, revealed the emergence of an extensively drug-resistant (XDR) variant that was not only resistant to the first-line antimicrobials chloramphenicol, ampicillin, and trimethoprim-sulfamethoxazole, but also fluoroquinolones and third-generation cephalosporins [120]. This variant, which was within the H58 clade, had acquired a plasmid encoding additional resistance elements, including a *bla*_CTX-M-15_ and a *qnrS* fluoroquinolone resistance gene [121].

Following the initial reports of XDR typhoid, intensified surveillance was undertaken to monitor its spread of typhoid into Karachi and beyond [122]. Reactive vaccination campaigns were initiated in Hyderabad and Karachi, and vaccine safety and effectiveness data were generated [123, 124]. All of these efforts played a major role in the decision of the Federal Expanded Program on Immunization to introduce typhoid conjugate vaccine (TCV) into their national immunisation programme, making Pakistan the first country to do so [26, 125]. The phased introduction began with a vaccination campaign targeting ~ 10 million children in the urban areas of Sindh province in November 2019, which were the areas hardest hit by XDR typhoid, and a subsequent campaign was conducted in Islamabad and Punjab province, where XDR had spread, that covered over 13 million children [126]. Consequently, WGS and phenotypic AMR data had a direct impact on the decision to introduce TCV and on the vaccine introduction strategy itself.

In addition to elucidating the molecular mechanisms of AMR and providing well-defined, standardised AMR data where mechanisms of resistance are known, WGS data can also provide important information regarding typhoid transmission pathways. The 2018 WHO Position Paper on typhoid vaccines indicated that TCV introduction should be prioritised “in countries with the highest burden of typhoid disease or a high burden of antimicrobial-resistant *S*. Typhi” [127]. Given the ubiquitous nature of drug-resistant typhoid, the anticipated country demand for TCVs, and the presence of only two WHO prequalified manufacturers of TCV, additional selection criteria may be required [127]. Globally representative WGS data show that drug-resistant typhoid typically emerges and spreads from South Asia [117]; therefore, prioritising TCV introduction in this region may reduce the burden of AMR in the region and prevent the spread of new resistance phenotypes internationally. Such phylodynamic data can inform optimal vaccine introduction strategies for other key highly drug-resistant pathogens, as and when new vaccines become available.

## Limitations, challenges, and future directions

### Technical limitations

The concept of relatedness between bacterial isolates is based on a process of continuous evolution, but prospectively identifying genetic signals of AMR evolution from the background noise of genetic variation and sequencing error is difficult. Using sequence data to infer relatedness and transmission is highly variable, and mutation rates can vary because of different lifestyles/conditions such as biofilm formation, antimicrobial exposure, disease states, and environmental pressures such as starvation [128–130]. The threshold number of SNPs above which relatedness is unlikely is therefore highly context-dependent [21, 80, 131]; if not recognised, then selection biases within isolates in collections can lead to false epidemiological inferences. Despite these challenges, defined cut-off points for the relatedness of specific pathogens, such as MRSA and *M. tuberculosis*, have been suggested in order to establish a threshold beyond which recent transmission is unlikely [132].

Inferring or excluding transmission events for pathogens that accumulate mutations slowly can be difficult [133], which presents a further challenge of differentiating genuine variants from sequencing error [134]. Interpreting the significance of WGS variation for pathogens that can establish asymptomatic carriage, have a period of latency, or cause a chronic, indolent illness can also prove challenging, as a degree of in-host genetic variation occurs with time, and different lineages may be intermittently transmitted at different points from a single host [13]. Bayesian mathematical approaches that integrate and model pathogen evolution with contact and temporal data on symptom onset, and that permit sequencing error, are being used to improve the inference of transmission chains, but understanding the sequence variance in real-world scenarios remains a major challenge [13, 133, 134].

Highly transmissible MGEs (such as plasmids) facilitate non-mutational AMR gene acquisition by HGT and are highly implicated in AMR dissemination [135]. In comparison, AMR that is driven by mutations on the bacterial chromosome is stable, and transmission is generally confined largely to progeny [136]. Determining the genetic location of AMR genes is therefore a key aspect of understanding AMR evolution and transmission, but is not readily determined by short-read sequencing [137–139]. Due to an inability to sequence long stretches of DNA and a failure to generate sufficient overlap in DNA fragments to allow accurate assembly of repetitive DNA sequences that are longer than the read length, these approaches are less well suited to describe the genetic environments of AMR genes [138, 139]. Additionally, AMR genes are commonly associated with long, repetitive insertion sequences, and short-read sequencing techniques cannot resolve if these are carried on a plasmid or chromosomally located [38, 54, 140, 141]. Most outbreak investigations have largely assumed the relative conservation of plasmid structures over the limited time period of an outbreak and the extent to which HGT events contribute to outbreaks of AMR is not fully understood [54]. However, more affordable long-read sequencing technology is becoming increasingly available and can generate reads that can span repetitive areas, allowing for the complete reconstruction of genome structure. This approach, however, is generally less accurate and has lower throughput compared to short-read sequencing [139, 140]. Hybrid approaches whereby isolates are sequenced en masse with short-read sequencing to determine identity, gene content, and relatedness, with selective addition of long-read sequencing to resolve the structure of MGEs, have been used to harness the benefits of both approaches [135, 139, 140, 142, 143].

### Challenges in implementing WGS for routine use

The routine, prospective inclusion of WGS into microbiological surveillance has the potential to greatly enhance and strengthen public health efforts to combat AMR but comes with significant logistical and financial implications [144]. Unfortunately, prospective data demonstrating and quantifying the beneficial impact of routine WGS implementation are limited [145, 146]; most data are derived from retrospective studies, specifically from studies reconstructing chains of transmission during outbreak investigations [145]. Cost-effectiveness is even harder to determine, reflecting marked uncertainties and challenges in estimating the costs for the implementation of WGS. Generating accurate, contemporary cost estimates is difficult in the context of the marked decrease in costs that has accompanied the rapid technological advances in recent years, a problem compounded by the challenge of estimating costs for complex workflows that include several steps, including downstream analysis [147]. Further, routine diagnostics frequently requires a rapid turnaround time to support the management of individual cases, necessitating the processing of individual samples. This approach contrasts with many research and surveillance studies that lend themselves to batch processing of samples [147]. Despite these challenges, studies showing the cost-effectiveness of WGS for infection control and outbreak detection and management are starting to emerge and will help support implementation decisions [148–151].

Ensuring the reproducibility and validity of results through standardisation and quality assurance (QA) processes is an essential part of routine medical laboratory processing [152]. However, implementing QA processes for WGS is challenging. Variations in DNA extraction methods and reagents, sequencing technologies, analysis pipelines, and bioinformatic approaches can all impact WGS analysis [152], making the standardisation and quality assurance methods challenging [147, 153]. Nevertheless, efforts at quality assurance and result standardisation across laboratories are currently being attempted for WGS workflows [154]. Ring trials showing high performance and consistency across laboratories of WGS methods applied to *S. aureus* are highly encouraging [152, 153] and suggest that developing isolate collections that can act as biological standards, against which reproducibility and robustness of methods can be measured, might be feasible.

The ability to share, integrate, and compare the vast wealth of data derived from WGS across laboratories, settings, and time is key to harnessing the power of WGS for global AMR surveillance [4, 154]. Barriers to data sharing include the reluctance of academics to share data before publication (a process that is itself often slow and time-consuming), political sensitivities when competing interests are at play (for example tourism), legal and requirements to protect personal data [154], and the need for standardised methodological and analytical approaches to generate comparable data [155]. Databases such as GenBank facilitate sharing of existing genomes, but their utility is again compromised by a lack of data standardisation, as well as deficiencies in accompanying metadata [154]. The Global Microbial Identifier (GMI) initiative is in the early stages of trying to address these issues, aiming to develop standardised identification and characterisation approaches in order to form a global interactive network of genomic databases [154]. The absence of validated globally standardised systems for genomic typing, defining clusters and determining AMR genes, further intensifies the challenges of interpreting genomic data [4, 11, 147]. Clinical and epidemiological metadata are essential for public health solutions to AMR, but are associated with standardisation, computational, and technical challenges [154]. The collaborative approach to sharing of the SARS-CoV-2 genomic data demonstrates that such an approach is possible [156, 157], but to date, scalable solutions to genomic data sharing have remained elusive.

### Introducing WGS in LMICs

Despite AMR being a major problem in LMICs, there is currently limited understanding of AMR dynamics in these settings [22, 28] and a notable lack of sustained access to WGS surveillance [11]. The capital investment required to establish and maintain WGS platforms is substantial. Supply, procurement, and maintenance of WGS reagents and equipment can be highly challenging [11, 158], particularly, in locations with inconsistent supply chains, where short shelf-life consumables and long intervals between procurement and delivery may create wastage or significant project delays. Support for data analysis is also indispensable, along with investment in training and retention of people to develop and sustain the necessary proficiency. Even simple analyses of bacterial WGS data require access to high-performance compute (HPC) clusters, stable internet access, and expertise [11].

## Conclusions

The rapid emergence and spread of AMR in recent decades are a major threat to global public health, and current antimicrobial development efforts cannot keep pace with pathogen evolutionary dynamics. Addressing this threat will require diverse, cross-sectoral interventions at all levels of public health and political systems. Improvements in genomic technologies in recent years have made these systems more widely accessible and affordable, and they are now being implemented as front-line tools in the battle against AMR. By enhancing AMR surveillance, WGS has greatly improved understanding of how, when, where, and why AMR emerges and spreads and has helped quantify temporal and geographical variations in AMR epidemiology. Genomic insights have also been applied to facilitate outbreak detection and control, improve diagnostic tests, optimise antimicrobial use, and inform vaccination strategies. Although logistical and technological barriers to the universal implementation of genomics in public health remain (particularly in LMICs), the use of these approaches is likely to further expand in the coming years and will hopefully help restrict the adverse public health impacts of AMR.

## Data Availability

Not applicable
